# The Sanitary Condition of the Laboring Population of New-York, with Suggestions for Its Improvement

**Published:** 1845-08-01

**Authors:** 


*The Sanitary condition of the Laboring population of New-York, with Suggestions for its improvement; by John H. Griscom, M. D.*—We have been gratified by the perusal of a pamphlet bearing the above title. Its objects are to portray the domiciliary condition of a large portion of the laboring classes in the city of New-York, especially emigrants from foreign countries; to show the connection which subsists between the circumstances surrounding their abodes and mode of living, and their moral and physical condition—the liability to disease, the low average of longevity and the proneness to vice; and to suggest improvements in the organization of the health police, in order to remedy and prevent the evils which now exist.

The author has treated the subject in a plain, perspicuous and forcible manner. The subject is one of great importance, and one which comes peculiarly within the province of medical philanthropy. It is needless to say that the duties of the medical practitioner are not restricted to the mere management of disease, but embrace the employment of the means which his knowledge and influence place at his disposal, to enlighten the community on the laws of vital existence and health, thereby preventing disease, with its accompanying trials and evils. Dr. Griscom appears to have taken pains to inform himself of the actual extent to which the conditions of health and longevity are violated among the laboring population in New-York, by the want of ventilation, close congregation in small apartments, neglect of cleanliness, insufficient drainage and sewerage, and the habitation of damp basements and cellars. He fortifies his conclusions of the pernicious influences of these, by the comparative duration of life, as derived from vital statistics in commercial and manufacturing towns, and the population of rural districts. We regret that our limits do not enable us to give some of the detailed results of his investigations. The evils of which he complains are by no means peculiar to New-York, or to large cities, but exist measurably everywhere, owing, as we believe, chiefly to the general inability to know and appreciate the principles which are involved in the preservation of health, and the prophylaxis of disease. We are not prepared to agree with Dr. G. that municipal regulations, and the supervision of a health police, are appropriate methods of improvement. The diffusion of the knowledge of Physiological laws and the conditions of healthful existence, seem to us to furnish the only efficient and permanent basis for a radical amelioration. When this obtains, public sentiment, and the instinct of self preservation will be adequate to the enforcement of proper sanitary measures, in so far as anything can prevail over human recklessness, and human depravity.

